# Bilateral nongranulomatous anterior uveitis associated with chancre of the tongue: initial presentation of syphilis

**DOI:** 10.1186/1869-5760-3-33

**Published:** 2013-02-11

**Authors:** Clovis Arcoverde Freitas-Neto, Vinícius Monteiro Castro, Daniel Vitor Vasconcelos-Santos

**Affiliations:** 1 Hospital de Olhos Santa Luzia, Estrada do Encanamento 909, CEP 52070-000, Recife-Pernambuco, Brazil; 2 Centro Brasileiro de Ciências Visuais, Belo Horizonte, Brazil; 3 Universidade Federal de Minas Gerais, Belo Horizonte, Brazil

**Keywords:** Ocular syphilis, Anterior uveitis, Tongue chancre, Penicillin, Case reports

## Abstract

**Background:**

This paper reports the case of a pleomorphic manifestation of syphilis, a reemerging sexually transmitted disease.

**Findings:**

A 51-year-old male presented with bilateral nongranulomatous anterior uveitis associated with an isolated syphilitic chancre of the tongue that was successfully treated with parenteral benzathine penicillin.

**Conclusion:**

Syphilis has the potential to lead to any type of intraocular inflammation. A careful review of the symptoms is useful for the detection of extraocular signs of syphilitic infection.

## Findings

### Introduction

Data from the World Health Organization indicate an increase in the incidence of syphilis, with an estimated 12 million new cases per year worldwide, particularly involving co-infection with human immunodeficiency virus (HIV) [1]. Ophthalmologists have the opportunity to diagnose and perform early treatment of this reemerging disease [2-4].

Ocular manifestations are reported in only 14% of immunocompetent individuals with syphilis. In those with HIV co-infection, however, this rate reaches 36% [5]. Posterior uveitis is the most common manifestation of syphilitic intraocular inflammation [6]. While serological tests are required to arrive at a definitive diagnosis, the recognition of other systemic signs of syphilis may also be helpful.

This paper describes a case of bilateral nongranulomatous anterior uveitis associated with a syphilitic chancre of the tongue.

### Case report

**Figure 1:**
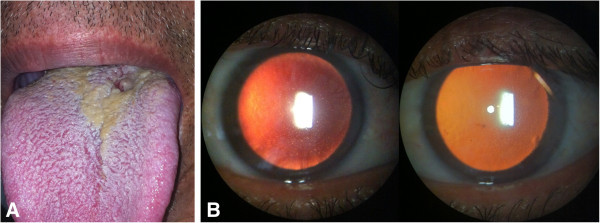
**Examination at presentation.** (**A**) Lesion consistent with syphilitic chancre on base of tongue; (**B**) slit lamp examination revealed small keratic precipitates and fibrin deposits overlying corneal endothelium in retroillumination of right and left eyes, respectively.

A 51-year-old man presented with a two-month history of pain, redness, and decreased vision in both eyes. The patient also reported a painless ulcerated lesion with hard edges on the base of the tongue, consistent with a chancre (Figure 1). Clinical examination also revealed left cervical lymphadenopathy.

The patient’s best-corrected visual acuity was 20/32 in the right eye and 20/25 in the left eye. Slit lamp examination revealed sparse fine keratic precipitates in both eyes, 2+ cells, and flare in the anterior chamber of the right eye and trace cells in the left eye. Dilated bilateral fundus examination was normal. Applanation tonometry revealed intraocular pressure of 26 mmHg in the right eye and 27 mmHg in the left.

The patient was submitted to a systemic workup, including complete blood count and screening tests for HIV, syphilis, and tuberculosis. Computer-assisted tomography of the head and a chest X-ray were also performed. All results were negative, except the syphilis serology, which revealed a positive venereal disease research laboratory (VDRL) test (titer of 1:128) and *Treponema pallidum* hemagglutination test. Lumbar puncture was recommended but could not be performed.

**Figure 2:**
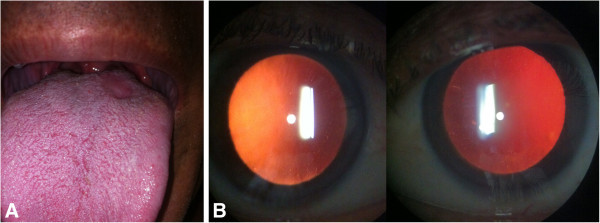
**Examination 4 weeks after presentation, following completion of treatment for syphilis.** (**A**) Tongue chancre healed; (**B**) slit lamp examination revealed resolution of signs of intraocular inflammation.

The patient was treated with aqueous crystalline penicillin G (4 million units intravenously every 4 h for 10 days) followed by benzathine penicillin (2.4 million units intramuscularly weekly for 3 weeks). Topical steroids in a tapering regimen, cycloplegics, and timolol maleate were also prescribed. After 4 weeks of antibiotic treatment, best-corrected visual acuity improved to 20/20 in both eyes, intraocular pressure decreased to 10 mmHg in each eye, intraocular inflammation was resolved and the tongue lesion had healed (Figure 2).

## Discussion

Syphilis is well known for its diversity of ocular and extraocular manifestations. Although oral ulcers have been widely recognized as possibly related to syphilitic infection, the occurrence of lesions consistent with an oral syphilitic chancre has not yet been clearly associated with intraocular inflammation [7-9]. Such lesions may be overlooked, as they are generally associated with primary syphilis and may spontaneously resolve before the development of ocular disease. Moreover, these lesions are not typically painful [10] and ophthalmologists do not routinely perform an examination of the oral cavity.

As no cerebrospinal fluid analysis could be performed in the present case, the patient received treatment for neurosyphilis despite the absence of retinal/optic nerve involvement, with the subsequent resolution of intraocular inflammation and healing of the tongue chancre.

## Conclusion

In view of the increasing incidence of syphilis, its association with HIV infection, and the variety of clinical manifestations, this sexually transmitted disease should be considered even in the setting of apparently innocent nongranulomatous anterior uveitis. Supportive systemic findings, which may include syphilitic chancre, should be investigated and are easily detected upon careful examination.

### Consent

Written informed consent was obtained from the patient for publication of this report and accompanying images. This work was performed with the approval of the Department of Ophthalmology of the Hospital das Clínicas de Minas Gerais (Brazil), according to the tenets of the declaration of Helsinki.

## Competing interests

The authors declare that there are no competing interests.

## Authors’ contributions

CF and VC contributed to the conception, design, data acquisition, and drafting of the article. DV was responsible for the conception and design, as well as for critically reviewing the intellectual content of the article. All authors read and approved the final manuscript.
